# Assessment of selected media supplements to improve F/HN lentiviral vector production yields

**DOI:** 10.1038/s41598-017-07893-3

**Published:** 2017-08-31

**Authors:** Jean-François Gélinas, Lee A. Davies, Deborah R. Gill, Stephen C. Hyde

**Affiliations:** 1 Gene Medicine Research Group, NDCLS, Radcliffe Department of Medicine, John Radcliffe Hospital, Oxford University, Oxford, UK; 2 United Kingdom Cystic Fibrosis Gene Therapy Consortium, Oxford, Edinburgh, London UK

## Abstract

The development of lentiviral-based therapeutics is challenged by the high cost of current Good Manufacturing Practices (cGMP) production. Lentiviruses are enveloped viruses that capture a portion of the host cell membrane during budding, which then constitutes part of the virus particle. This process might lead to lipid and protein depletion in the cell membrane and affect cell viability. Furthermore, growth in suspension also causes stresses that can affect virus production yields. To assess the impact of these issues, selected supplements (Cholesterol Lipid Concentrate, Chemically Defined Lipid Concentrate, Lipid Mixture 1, Gelatin Peptone N3, N-Acetyl-L-Cysteine and Pluronic F-68) were assayed in order to improve production yields in a transient transfection production of a Sendai virus F/HN-pseudotyped HIV-1-based third generation lentiviral vector in FreeStyle 293 (serum-free media) in suspension. None of the supplements tested had a significant positive impact on lentiviral vector yields, but small non-significant improvements could be combined to increase vector production in a cell line where other conditions have been optimised.

## Introduction

The use of viral vectors for therapeutic gene delivery capitalises on the co-evolution of viruses and mammalian host cells^1^ that naturally makes them efficient gene transfer agents. A number of naturally-occurring viruses have been adapted as viral vectors for gene therapy^2^. Lentiviruses are particularly suited for this purpose because they can integrate into the host genome, have a large transgene capacity and can transduce both dividing and non-dividing cells^3^. Therapeutic lentiviral vectors have encountered pre-clinical success in *ex vivo* clinical trials for treatment of leukaemia^4–6^ and using haematopoietic stem cells^7–10^ and are being assayed in early *in vivo* clinical trials for Parkinson’s disease^11^ and age-related macular degeneration^12^.

The United Kingdom Cystic Fibrosis Gene Therapy Consortium is currently developing a lentiviral vector platform which is pseudotyped with the Sendai virus (murine parainfluenza virus type 1) fusion and haemagglutinin-neuraminidase (F/HN) envelope proteins^13^, to facilitate delivery to lung cells^14, 15^. In Sendai virus, the HN protein is responsible for haemagglutination and receptor binding and has neuraminidase activity^16^. The glycoprotein F is required for virus-induced haemolysis, cell fusion and the initiation of infection^17^. This lentiviral vector product is now being considered for current Good Manufacturing Practice (cGMP) vector production and clinical evaluation.

The development of lentiviral-based therapeutics is hindered by the high cost of cGMP production. This is particularly relevant for *in vivo* applications where large quantities of viral vector may be required, as opposed to the smaller quantities sufficient for *ex vivo* transduction. Furthermore, production titres for lentiviral vectors appear several log-orders lower than those typically obtained for recombinant Adenoviral and Adeno-Associated Viral vectors, which have benefited from improvements in vector production in recent years^18, 19^.

Production of viral gene therapy products often involves the use of bovine serum in cell culture. This supplementation is performed in order to provide a source of essential nutrients to the cells and increase virus production yields. The use of serum in cell culture poses ethical considerations^20^ as well as a potential risk of transfer of viruses or prions into the final product^21^. While extensive contaminant testing could address the safety issue, it can be mitigated by careful tracking of the reagents’ sources (i.e. country of origin, batch number, etc.). It does, however, remain a risk, which must be assessed and reported^22^. Furthermore, immune responses caused by the use of bovine serum have been observed in bone marrow transplant^23^, cell therapy^24, 25^ and gene therapy^26^ clinical trials. As a result, there is regulatory pressure to reduce or remove animal serum from production processes^21^. Moreover, if serum continues to be used, supply limitation of certified material is another issue which could arise, with demand rising as more products reach the stage of large-scale manufacture^27^. While human serum in cell culture^28^ could be used as an alternative for small-scale manufacturing, it is unlikely to be a viable option, in terms of both the supply and cost, for long-term, large-scale manufacturing.

Lentiviral vectors have been produced in the absence of serum, reducing the immunogenicity of the final product without affecting final transduction efficiency^29^. In turn, efforts to reduce the reliance on serum, and to provide more completely defined mammalian cell growth conditions, have led to the development of specialised media that are both serum-free and protein-free. One popular growth medium for lentivirus production is the FreeStyle™ 293 Expression Medium (FreeStyle 293)^30^. It is a chemically defined, animal origin-free, protein-free medium developed to support growth and transfection of FreeStyle™ 293-F cells, a fast-growing clonal isolate of HEK 293 that has been adapted to suspension culture in serum-free media. FreeStyle 293 is manufactured at a cGMP-compliant facility and is marketed as suitable for cGMP production of vectors. It contains the GlutaMAX™ supplement, marketed as minimising toxic ammonia build-up and improving cell viability; however, the rest of its formulation is proprietary. It is therefore not obvious to assess if constituents usually present in serum added as a supplement might be lacking for optimised lentiviral vector production.

Serum deprivation during vector production was shown to affect producer cells. Depending on the cell type, it can lead to reduction in total lipid content and, therefore, reduction in infectious vector titres, while in other cell types it will lead to a rise in *de novo* lipid biosynthesis, particularly for cholesterol, reducing the impact on titres^31^. In addition, lentiviruses being enveloped viruses, when newly developed virions bud from producer cells they capture a portion of the cell membrane, which then constitutes a key component of the viral particle. Viral and host cell membranes are of different composition; the HIV-1 envelope is, for example, more ordered than the host cell membrane from which it originated^32^. HIV-1 buds from areas of the plasma membrane with high levels of lipid rafts^33^ which are organised areas enriched in cholesterol, sphingolipids, and glycosylphosphatidylinositol-linked proteins^34^. It has been hypothesised that these components are preferentially incorporated in the viral envelope as a result of preferential sorting of HIV-1 Gag to lipid rafts^33^. More specifically, the molar ratio of cholesterol to phospholipid is about 2.5 times higher in virion envelopes compared to the host cell membrane^32^ and the levels of several other raft lipids and proteins are elevated in virions compared with their progenitor cells. We hypothesise, therefore, that the exodus of proteins and lipids that occurs during virus budding could deplete the producer cells’ reserves, and ultimately reduce viability, leading to a lower rate of virus production. Furthermore, growth in suspension culture is associated with stresses due to shear forces and bubble-bursting which can also affect production yield^35^. To begin to address these issues, the addition of different media supplements to the producer cell growth medium was explored for the potential to increase lentiviral vector production titres in the specific context of the Sendai virus F/HN-pseudotyped lentiviral vectors.

## Results

### Standardisation of lentiviral vector production

The production of a third generation HIV-1-based lentiviral vector, expressing an Enhanced Green Fluorescence Protein (EGFP) and Firefly Luciferase (Lux) fusion protein (EGFPLux) was evaluated in the presence of a variety of supplements. This vector was pseudotyped with the Sendai virus F/HN envelope proteins^13^ to generate rHIV.F/HN CMV- EGFPLux. The first step to accomplish this was to develop an experimental method that could result in reproducible lentivirus yields.

**Figure 1:**
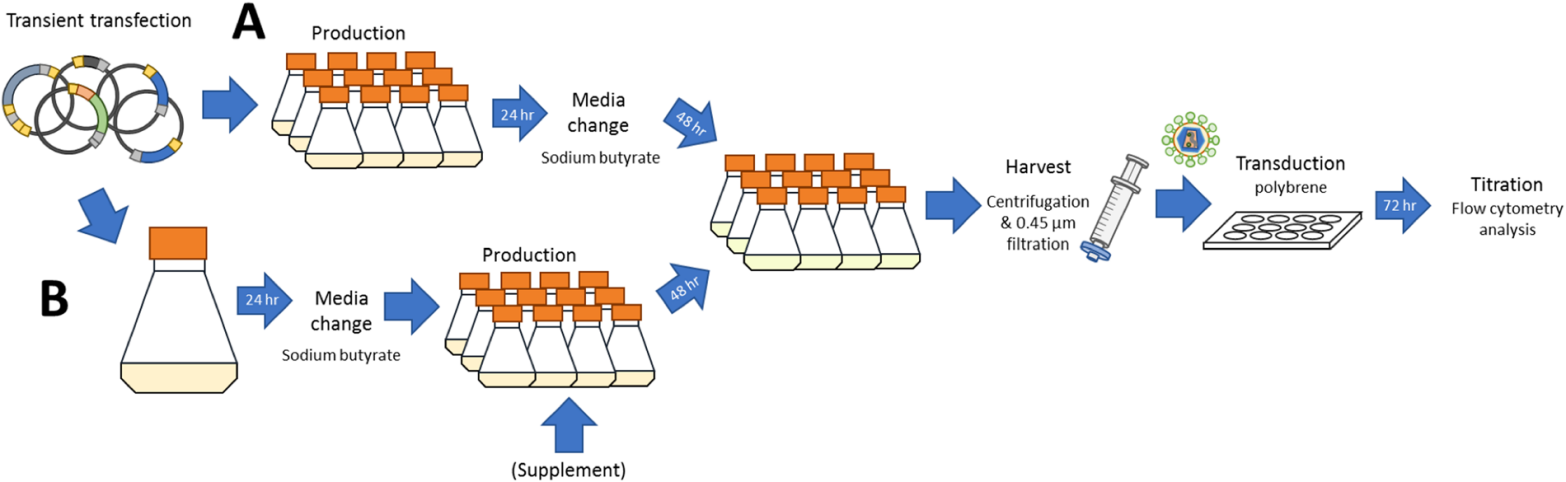
Schematic representation of the different transient transfection, production and titration protocols for lentiviral vectors produced in flasks. In both cases, Human Embryonic Kidney 293T cells (HEK 293T) were transiently transfected with a ‘transfection mix’ of the five plasmids required for virus production complexed with polyethylenimine (PEI). (**A**) In the multiple independent transfections strategy, cells were split into the required number of replicates prior to transfection and the ‘transfection mixes’ were prepared independently for each flask. Media change was performed after 24 hours including the addition of sodium butyrate. (**B**) In the single-flask transfection strategy, the cells were split after transient transfection into the required number of replicates. Media change and sodium butyrate addition were performed after 24 hours and, if appropriate, the assayed supplement added at this point. (**A** and **B**) After an additional 48 hours of incubation, the production was harvested by centrifugation and the supernatant filtered. A sample of the filtrate was used for transduction in a 12 well plate seeded with HEK 293T in media containing hexadimethrine bromide (polybrene) After a 72-hour incubation, the titre was determined by flow cytometry.

Preliminary studies indicated that the transient transfection step was a large source of the variability between vector production experiments. Hence, the impact on production variability of two alternate transfection strategies was evaluated: a multiple independent transfections strategy, where 12 flasks of cells were transfected independently (Fig. 1A), and a single-flask transfection strategy, where cells in a single, larger seed flask were collectively transfected and then subsequently split into 12 smaller flasks (Fig. 1B).

**Figure 2:**
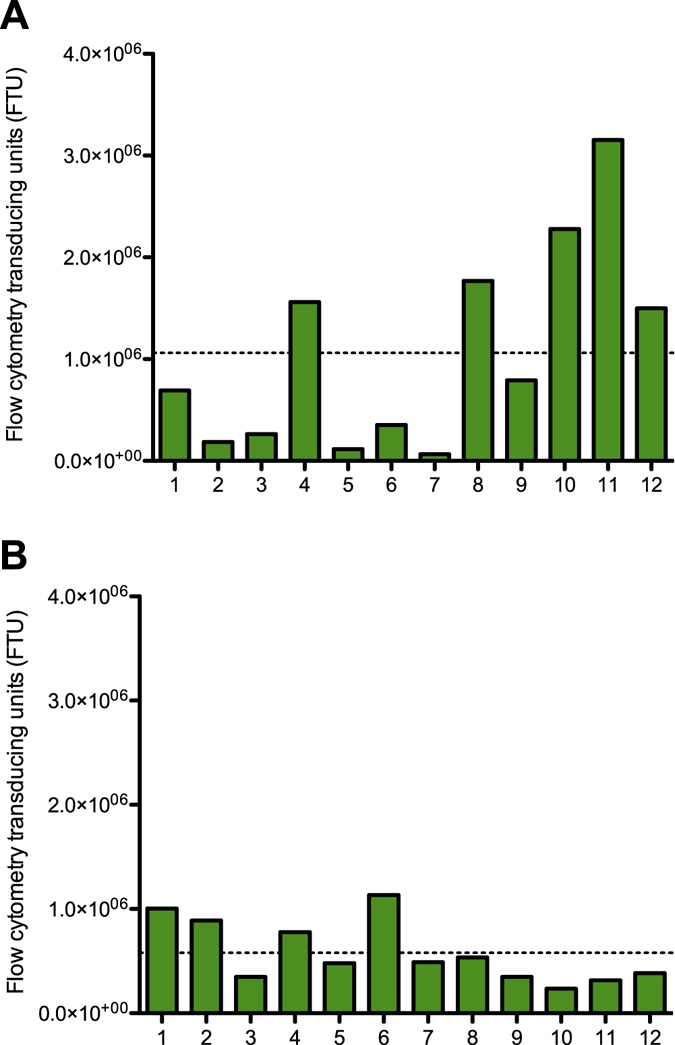
Lentiviral vector production yields for the multiple independent transfections strategy flask and the single-flask transfection strategy. Individual rHIV.F/HN CMV- EGFPLux yields from flasks containing non-supplemented medium from (**A**) multiple independent transfections strategy (12 independent 125 mL flasks at 1 × 10^6^ HEK 293T cells/mL in 20 mL) where cells were transfected with independently prepared ‘transfection mixes’ necessary to produce rHIV.F/HN CMV- EGFPLux, or (**B**) single-flask transfection strategy where HEK 293T cells (1 × 10^6^ cells/mL in a 1000 mL flask) were transfected with the five plasmids necessary to produce rHIV.F/HN CMV- EGFPLux and subsequently divided into 12 separate 20 mL cultures in 125 mL flasks. Titres were measured by flow cytometry (flow cytometry transducing units: FTU/mL). The dashed lines show the average titre for all flasks for each strategy.

For the multiple independent transfections strategy, 20 mL of HEK 293T cells at 1 × 10^6^ cells/mL were seeded in 12 separate 125 mL flasks. Twelve independently prepared transfection mixtures (each comprising 30 μg of plasmid DNA) were used to transfect the flasks. The media was changed after 24 hours and the rHIV.F/HN CMV- EGFPLux virus generated was harvested after a further 48 hours’ incubation. The viral titre for each of the 12 independent cultures, as determined by flow cytometry, is shown in Fig. 2A.

For the single-flask transfection strategy, a single ‘transfection mix’ was prepared for transfection of 240 mL of HEK 293T cells at 1 × 10^6^ cells/mL seeded into one 1000 mL flask with 360 µg of the vector plasmid DNA mixture. Twenty-four hours after transfection, the media was changed by centrifugation and the culture divided between 12 separate 125 mL flasks, 20 mL per flask. The rHIV.F/HN CMV- EGFPLux virus generated was harvested after a further 48 hours’ incubation. The virus titre for each flask was determined using flow cytometry and results are shown in Fig. 2B.

As shown in Fig. 2A and B, the absolute titres of rHIV.F/HN CMV- EGFPLux generated by these two approaches vary largely between replicates. To be able to compare the performance of different supplements, titres were normalised to the average titre of the non-supplemented flasks in each experiment. Comparing the normalised titration results from the multiple independent transfections strategy (mean of twelve flasks: 100, standard deviation: 93.5) with those from the single-flask transfection strategy (mean of twelve flasks: 100, standard deviation: 51.4) indicated that there was less variation observed using the single-flask transfection strategy. Using these data, to model future studies incorporating three replicates, statistical power analysis demonstrated the superiority of the single-flask transfection strategy. Using the multiple independent transfections approach a minimum of a 3.33-fold increase in titre would be necessary to observe a statistical difference with 80% power using triplicates, whereas the single flask approach could readily distinguish a 2.28-fold increase in titre with the same power. Given the reduced variability, and the anticipated lower threshold for reaching statistical differences when comparing experimental groups, the single-flask transfection strategy was selected for all subsequent experiments.

### Cholesterol Lipid Concentrate supplementation does not improve yields

To address the possibility of lipid depletion in cells producing lentiviral vectors, several lipid supplements have been investigated with the aim of increasing lentiviral vector productivity and/or infectivity. Of these, cholesterol-based supplements have been shown previously to significantly improve production titres of Vesicular Stomatitis Virus G glycoprotein (VSV-g)-pseudotyped lentiviral vectors in the presence of serum^36, 37^ and to restore infectivity in serum-deprived retroviral vector production to levels seen when serum was used^38^. The timing of lipid supplementation is crucial as addition prior to transfection can profoundly decrease viral yields (Figure S1). One potential explanation for this unexpected adverse effect of lipid supplementation was that it had an adverse effect on HEK 293T cell transfection with the producer plasmid mixture. To mitigate the impact of this potential effect, a study was performed where Cholesterol Lipid Concentrate (CLC), a commercial, animal-free, cholesterol-based proprietary formulation sold as a 250x concentrate suitable for cGMP manufacturing supplementation was carried out during rHIV.F/HN CMV- EGFPLux production 24 hours post-transfection.

**Figure 3:**
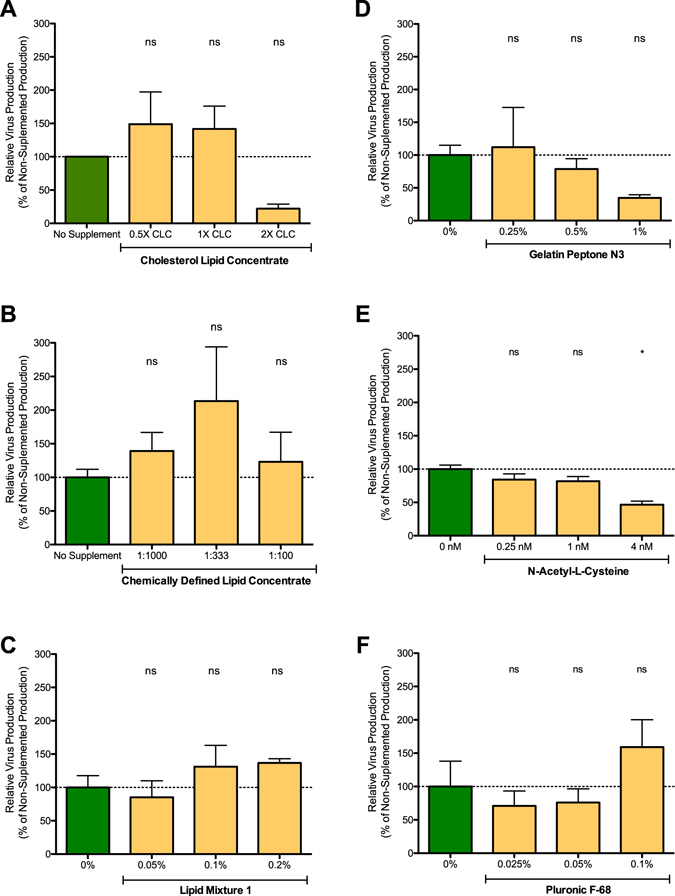
Lentiviral vector titres following lipid medium supplementation. Yields of rHIV.F/HN CMV- EGFPLux in non-supplemented medium (dark green) or medium supplemented (yellow) with (**A**) Cholesterol Lipid Concentrate, (**B**) Chemically Defined Lipid Concentrate or (**C**) Lipid Mixture 1, (**D**) Gelatin Peptone N3, (**E**) N-acetyl-cysteine, or (**F**) Pluronic F-68. Titres were measured by flow cytometry and are shown relative to non-supplemented values. Bars represent the mean of triplicate production studies ± standard error of the mean. There were no statistically significant differences between group means as determined by one-way ANOVA, except in (E): (**A**) F(3,8) = 3.798, p = 0.0582, (**B**) F(3,8) = 1.025, p = 0.4315, (**C**) F(3,8) = 1.230, p = 0.3606), (**D**) F(3,8) = 1.113, p = 0.3993, (**E**) F(3,8) = 11.04, p = 0.0032 and (**F**) F(3,8) = 1.621, p = 0.2598, followed, if appropriate, by Dunnett’s post-test. A calculated p value of > 0.05 was deemed non-significant as indicated with ns on charts. A calculated p value of < 0.05 was deemed a significant difference as indicated with one star (*) on charts.

Post-transfection CLC supplementation was investigated using the single-flask transfection strategy described above. The steps for supplementation studies are presented in Fig. 1B. A single 1000 mL flask containing 1 × 10^6^ cells/mL in 240 mL media was transfected with 360 µg of the plasmid mixture required to produce rHIV.F/HN CMV- EGFPLux. Twenty-four hours after transfection, the flasks were centrifuged for media change and the pelleted cells resuspended in 225 mL FreeStyle 293 with sodium butyrate. The culture was then divided between 12 separate 125 mL flasks (18 mL per flask). Triplicate flasks were supplemented with 2 mL of CLC diluted in FreeStyle 293 for final concentrations of 0.5X, 1X or 2X CLC in a final volume of 20 mL. Three flasks, where 2 mL of FreeStyle 293 without CLC were added, served as a triplicate negative control. Virus produced under these conditions was harvested 48 hours after supplementation and titrated. It is relevant to note that some of the supplement in the production media is carried over to the titration plates. It is not, however, expected to have a significant effect as the volume of production supernatant used for titration represents only about 0.5% of the volume in the titration wells. Contrary to lipid supplementation before transfection, here, there was no significant change in titre with any of the CLC concentrations evaluated post-transfection (Fig. 3A), although a trend for inhibition of virus production was noted with 2x CLC.

Methyl-beta-cyclodextrin (mβCD) is used as a complexing agent in the CLC formulation to enhance solubility in water-based tissue culture media^39^. Interestingly, mβCD can selectively extract cholesterol from the plasma membrane^40, 41^, which can affect cellular processes such as endocytosis^42^ as well as cell sensitivity to HIV-1 infection^43, 44^. A number of studies have explored the inhibitory impact of mβCD on HIV-1 yields^45^ and infectivity^46, 47^. This impact appears less when pseudotyping vectors with VSV-g^47^, although the effect, if any, on a Sendai virus F/HN pseudotype (as used here) is unknown. A possible solution to this problem would be to exclude mβCD from the cholesterol supplement, however, the CLC manufacturer was unable to produce either a mβCD-free CLC or CLC-supplemented medium, (Life Technologies, personal communication). It was, therefore, determined that CLC could not be used as a possible supplement at this stage.

### Chemically Defined Lipid Concentrate supplementation does not significantly improve yields

Due to the possible issues of mβCD-containing supplements, an alternative mβCD-free supplement containing cholesterol, Chemically Defined Lipid Concentrate (CDLC), was evaluated. CDLC is a concentrated lipid emulsion of saturated and unsaturated fatty acids and surfactants, designed to reduce or replace foetal bovine serum in cell culture media. It has been shown to improved titres of VSV-g-pseudotyped lentiviral vectors in the presence of serum^36^ and restore infectious particle productivity of retroviral vectors to the levels seen when using serum in HEK 293-derived cells^38^. The impact of CDLC addition to rHIV.F/HN CMV- EGFPLux production using the single-flask strategy was evaluated. A single 1000 mL flask containing 1 × 10^6^ cells/mL in 240 mL media was transfected with 360 µg of the plasmid mixture required to produce rHIV.F/HN CMV- EGFPLux. Twenty-four hours after transfection, the flasks were centrifuged for media change and the cells resuspended in 225 mL FreeStyle 293 with sodium butyrate. The culture was then divided between 12 separate 125 mL flasks (18 mL per flask). CDLC is supplied as a concentrate and the manufacturer’s guidelines recommended evaluating dilutions ranging from 1:100 to 1:1000. Triplicate flasks were supplemented with 2 mL of CDLC diluted in FreeStyle 293 for final dilutions of 1:1000, 1:333 or 1:100 of CDLC in a final volume of 20 mL. Three flasks, where 2 mL of FreeStyle were added without CDLC, served as a triplicate negative control. Virus produced under these conditions was harvested 48 hours after supplementation and titrated. Although a trend for increased virus production in the presence of CDLC was observed, there was no significant change in titres with any of the CDLC concentrations evaluated (Fig. 3B).

### Lipid Mixture 1 supplementation does not improve yields

It has been reported that Lipid Mixture 1 can be used as a supplement in a VSV-g pseudotyped lentiviral vector producer cell line grown in a low-calcium, serum-free media^48^. According to the manufacturer, Lipid Mixture 1 contains non-animal derived fatty acids (2 μg/mL arachidonic and 10 μg/mL each linoleic, linolenic, myristic, oleic, palmitic and stearic), 0.22 mg/mL cholesterol from New Zealand sheep’s wool, 2.2 mg/mL Tween-80, 70 μg/mL tocopherol acetate and 100 mg/mL Pluronic F-68 solubilised in cell culture water. Lipid Mixture 1 has been shown to increase 25 kDa linear polyethylenimine (PEI) transient transfection efficiency and, when used at 0.1% v/v, to not affect cell growth in HEK 293 derived cells^49^. The impact of Lipid Mixture 1 on lentiviral vector production yields was therefore assessed here. As described above, a single, large flask of transfected cells was prepared and, 24 hours later, split into 12 flasks after the media change, at which point sodium butyrate was added. Triplicate flasks were supplemented with 0.5X (0.05%), 1X (0.1%) or 2X (0.2%) the reported beneficial concentration of Lipid Mixture 1, or with FreeStyle 293 only, as a negative control. Virus produced under these conditions was harvested 48 hours after supplementation and titrated. Figure 3C shows that none of the concentrations had a significant impact on virus titre.

### Gelatin Peptone N3 supplementation does not improve yields

Gelatin Peptone N3 is also used as a supplement in a VSV-g pseudotyped lentiviral vector producer cell line grown in a low-calcium serum-free media^48^. Gelatin Peptone N3 is manufactured by a controlled enzymatic hydrolysis of gelatine and has been shown to have a positive effect on cell growth and transfection efficiency in HEK 293 derived cells at a concentration of 0.5% v/v^49^. The impact of Gelatin Peptone N3 on production yields was therefore assessed. A single large flask of transfected cells was prepared and, 24 hours later, split into 12 flasks after the media change, at which point sodium butyrate was added. Triplicate flasks were supplemented with half (0.25%), once (0.5%) or twice (1%) the optimal reported concentration, or with FreeStyle 293 only, as a negative control. Virus produced under these conditions was harvested 48 hours after supplementation and titrated. Figure 3D shows that none of the concentrations of Gelatin Peptone N3 tested had a significant impact on virus titre, although a trend for inhibition of virus production was noted with 1% supplementation.

### N-Acetyl-L-Cysteine supplementation does not improve yields

Medium supplements that are not membrane components, but that can still affect cell viability, were also assayed. N-Acetyl-L-Cysteine (NAC) is a widely used mucolytic agent and paracetamol overdose management medication with a general anti-apoptotic effect^50^ and is also known to have effects on HIV. Cell treatment with NAC can inhibit HIV-1 Long Terminal Repeat (LTR)-directed gene expression^51^, suggesting an inhibitory effect on HIV-1 transcription. NAC was therefore assayed as an anti-HIV drug, showing no benefit in asymptomatic patients^52^, but possible benefit if taken before initiating anti-retroviral therapy^53^. In cell culture HIV-1 replication studies, however, a dose-dependent effect of NAC has been observed on HIV-1 yields^54^. NAC doses of 0.12 and 0.25 mM decreased the infectious HIV-1 yield up to two-fold and cell viability to 75% of the level of non-supplemented controls. By contrast, NAC doses of 0.5–2 mM increased HIV-1 yield up to two-fold, while increasing cell viability to 120% of the level of non-supplemented controls, demonstrating a correlation with NAC’s modulation of cell growth. Finally, in chronically infected T lymphocytes, high NAC concentrations (4–16 mM) increased the cell proliferative rate increasing virus multiplication 4- to 6-fold, although, at such concentrations NAC also had an inhibitory effect in acutely infected cells, interfering with early events in the life cycle and reducing titres up to 4-fold^54^.

The impact of NAC on lentiviral vector production yields was therefore assessed. As described above, a single large flask of transfected cells was prepared and, 24 hours later, split into 12 flasks after the media change for supplementation. In an attempt to cover the reported range of concentrations affecting HIV-1 yields and cell proliferation^54^ three doses were assayed: 0.25 mM (reported to decrease HIV-1 yields), 1 mM (reported to increase HIV-1 yields) and 4 mM (reported to increase cell proliferation, but to decrease titres in acutely infected cells) as well as a FreeStyle 293 only control. Virus produced under these conditions was harvested 48 hours later and titrated. The results showed that, under the conditions used here, the two lower concentrations did not have a significant impact on virus production yield, contrary to previous reports using different conditions. However, the higher NAC dose had a significant (p < 0.05) two-fold adverse impact on virus titre (Fig. 3E) and consistent with NAC’s reported HIV-1 interference capacity at high doses in acutely infected cells^54^.

### Pluronic F-68 supplementation does not significantly improve yields

Polaxamers, such as Pluronic F-68, are non-ionic surfactants, comprising triblock copolymers made of hydrophilic polyethyleneoxyde and hydrophobic polypropylene oxide. Pluronic F-68, is widely used to protect cells from injury due to agitation and/or gas bubble sparging in bioreactors^35^. The cell protective effect of Pluronic F-68 has been attributed to two possible mechanisms: (i) it could reduce the cell-to-bubble attachment^55^ and damage caused by bubble bursting^56^, (ii) it could also increase the resistance of the cells to shear stress by decreasing the plasma membrane fluidity in cells^57^. Pluronic F-68 is known to be a component of FreeStyle 293, and the manufacturer’s recommendations suggest supplementation with an additional 2.5–5 mL/L of 10% Pluronic F-68 (0.025–0.05%) in large-scale bioreactor suspension cultures to reduce shear stress in the culture. While it was not assumed that additional Pluronic F-68 would necessarily increase viral titres in flask conditions, it was critical to rule out a deleterious effect on titres in bioreactors. An experiment was therefore designed to assay vector production yields using medium supplemented with additional Pluronic F-68.

As described above, a single, large flask of transfected cells was prepared and, 24 hours later, split into 12 flasks after the media change for supplementation, at which point sodium butyrate was added. The manufacturer’s recommended concentration of Pluronic F-68 supplementation is 0.05%, therefore, after the media change, triplicate flasks were supplemented with 0.5X (0.025%), 1X (0.05%) or 2X (0.1%) of the upper limit of the recommended concentration of additional Pluronic F-68, or with FreeStyle 293 only, as a negative control. Virus produced under these conditions was harvested 48 hours after supplementation and the virus titrated. Vector titres were not affected by any concentration of Pluronic F-68 added in the culture medium of the transfected cells (Fig. 3F), although a trend for enhanced virus production was noted with 0.1% Pluronic F-68.

## Discussion

A critical parameter affecting the cost of viral vector manufacturing is the final production titre, therefore, improvements to lentiviral vector production processes should ultimately increase efficiency and reduce overall costs. To this effect, serum-free media reduces the complexity, duration and cost of downstream processing as well as risks of transfer of viruses or prions into the final product. Furthermore, suspension production increases yields^58^ and allows the use of controlled bioreactors enhancing reproducibility. The experiments described here aimed to address issues arising during lentiviral vector production in serum-free suspension culture, specifically, the potential for depletion of cell membrane lipid and protein components that could occur due to high levels of virus production, as well as mitigating stresses due to bubble-bursting and shear forces generated during cell culture.

A simple protocol for a well-controlled, small-scale (20 mL) lentiviral vector production was developed based on transient transfection of HEK 293T cells in suspension. Several steps were taken to reduce potential variability, including the use of a master mix to bulk transfect cells in a single, large flask, which was then split to prepare 12 identical flasks of transfected cells for experimentation. This approach more closely resembles that adopted when using a stable producer cell line where all cultures are derived from a single, typically clonal, source. This allowed the experiments described here to be powered at 80%, sufficient to detect a 2.28-fold increase in titre.

Using this protocol, several media supplements were evaluated for their effect on lentiviral vector titre. All supplements tested were added 24-hour after transient transfection with the component lentiviral vector plasmids to minimise interaction of the supplement with the transfection mixture/process. Disappointingly, none of the media supplements tested significantly increased vector titre in these studies. CDLC supplementation led to the largest increase in titre (2.13-fold), but the observed trend did not reach significance, both because it did not reach the 2.28-fold threshold determined by power calculations and because of great inter-replicate variability (±0.81-fold). A follow-on study incorporating a greater number of replicates might confirm a modest increase in virus productivity with this approach. Another possibility would be to aggregate marginal gains by combining supplements with multiple non-significant (but positive) trends such as a combination of 1:333 CDLC and 0.1% Pluronic F-68.

To prevent any interference with the transfection process, supplements were always added at the media change step. It is possible, however, that a longer exposure of the cells to the supplements might be necessary to observe detectable effects in virus yield. Alternatively, it is also possible that the transfection step itself could be enhanced by the supplement as it has been reported for Lipid Mixture 1 and Gelatin Peptone N3^49^.

In the case of lipid supplements, the issue of the presence of mβCD in the supplements was noted above. It is also relevant to note the reactivity of mβCD with polyethylene which lines bioreactors used for large-scale production such as the WAVE Bioreactor (GE Healthcare)^30^. This issue was previously described^59^ following the unsuccessful growth of cholesterol-dependent cell lines in this type of bioreactor^60^. This would prevent the use of CLC in such bioreactors as it might cause leaching of cholesterol from the cell membrane. A possible solution would be to use an alternative bioreactor coating.

It is also possible that the increase in infectivity observed in VSV-g pseudotyped lentiviral vectors following cholesterol supplementation could not be reproduced here because of the different pseudotype. While mβCD-induced cholesterol depletion was shown not to influence VSV-g transport from the Golgi to the plasma membrane^61^, cholesterol depletion using statins has been shown to impair the transport of VSV-g from the endoplasmic reticulum to the Golgi and that cholesterol supplementation can reverse this impairment^62^. The effects of cholesterol depletion on Sendai virus F/HN migration to the cell surface have not been studied. Another solution to cholesterol depletion has recently been presented in which, instead of using a media supplement, a plasmid is transfected which leads to the overexpression of 3-hydroxy-3-methylglutaryl-coenzyme A reductase, a crucial cholesterogenic enzyme, in the producer cells^63^. This was reported to increase *de novo* cholesterol biosynthesis and to enhance by 2- to 3-fold both the physical and infectious titres.

In the case of polaxamers, Pluronic F-68 is reported to protect cells from sparging and injury due to agitation in bioreactors^35^. It is listed by the manufacturer as a component of FreeStyle 293 media, but at an undisclosed concentration. It is further recommended to supplement with an additional 2.5–5 mL/L of 10% Pluronic F-68 in agitating bioreactors. As no adverse effect was observed on titres in the experiment described above, it was decided to implement 5 mL/L 10% Pluronic F-68 (0.05%) supplementation in the ongoing large-scale production of lentiviral vectors)^30^. Each large-scale (1 L and 5 L) production run being unique, expensive and with multiple changing parameters, it has not been possible to determine the significance of any beneficial effect observed following this supplementation. However, cell counts and viability did appear to have increased and, importantly, no dramatic deleterious effects have been observed following implementation in bioreactors (data not shown).

Another supplement, chloroquine, has been extensively studied in the literature in this context. Chloroquine is a lysosomotropic amine used as an effective and safe anti-malarial and anti-rheumatoid agent. Its accumulation inhibits enzymes present in lysosomes and therefore proteolytic processes^64^. The inhibition of lysosomal enzymatic activity has also been shown to inhibit the degradation of DNA transfected with calcium phosphate^65^. This effect was observed in a calcium phosphate transient transfection production of retroviral vectors where titres where doubled with chloroquine addition^66^. However, the enhanced gene expression and transfection efficiency is counterbalanced by extensive cell toxicity after relatively brief (>4 hours)^67^ exposure. In large-scale production, quickly removing a toxic reagent is difficult and having to perform such a step would preferably be avoided.

While chloroquine has also been used in lentiviral vector production^36, 68–72^, a study of its effects on lentiviral yields detected no change in titres in a calcium phosphate transient transfection production^73^. Furthermore, the transfection reagent might be critical in chloroquine’s effect. Chloroquine addition was observed to reduce lentiviral vector titres when used in combination with PEI in DMEM without serum^74^. It has been suggested that PEI has a similar ‘DNA protection effect’ as chloroquine in lysosomes^75^, which might explain this discrepancy. For these reasons, chloroquine was not tested as a supplement here.

Several protein, lipid and other supplements are commercially available that could ultimately benefit lentiviral vector production, but to thoroughly evaluate these would probably require a protocol with a higher practical throughput. Moreover, the supplements tested in this study could have a benefit in an alternate experimental production setup. This is exemplified by the discordant results found with NAC supplementation in the present experimental setup compared to those obtained in HIV-1 infection of lymphocytes. It is therefore possible that third generation lentiviral vector production and the native HIV-1 life cycle have different requirements. Some supplements, such as caffeine, have been shown to increase lentiviral titres^76^, but have not been shown to be efficacious in the production system used in this study (data not shown). The use of the Sendai virus F/HN pseudotype might also result in different requirements compared with the more widely used VSV-g pseudotype. Both pseudotypes comprise proteins that are integrated into the cell membrane, but the exact effect of their presence on membrane composition is unknown.

The FreeStyle 293 media, used for the experiments described here, is a specialised medium developed for the optimal growth of HEK 293 derived cells in suspension and it is possible that only marginal benefit can be obtained by any additional media supplementation in this context. This could explain why supplements observed to improve production yields in other media have not been successful here. It may, therefore, be helpful to reassess the potential of these supplements to benefit production if alternative (perhaps non-optimal) media were used.

Finally, while variability was minimised using a single transfection rather than 12 independent transfections for each experiment, the variability of the experimental setup did not allow determination of small variations in titres (below 2.28-fold) as being significant. As described earlier in the case of CDLC, a larger number of replicates could have addressed this problem. An alternative setup involving a producer cell line could also be employed to minimise variation, removing the variability introduced by the transfection step.

Another factor that might explain the lack of effect of supplementation in the experimental setup, compared with an observed impact in other studies, might be sub-optimal production yields in the present setup. Even with a producer cell line, if the maximal production of lentiviral vector particles is not reached, then depletion of membrane components may not yet be rate-limiting. Thus it may be useful to reassess the possible benefit of such supplementation during late-stage production optimisation.

In summary, this study evaluated the addition of a variety of supplements to the producer cell growth medium, for the potential to increase lentiviral vector production titres. In the particular context of rHIV.F/HN CMV- EGFPLux production in FreeStyle 293 media, none of the supplements evaluated successfully enhanced vector production.

## Methods

### Cells

The HEK 293T/17 (HEK 293T)^66^ (ATCC, Manassas, VA, USA, CRL-11268) cell line was used in all experiments. It was adapted to growth in suspension using sequential serum reduction, accomplished by transitioning to an increasing proportion of new media by 25% per passage, with agitation starting when the transition was complete. The cells were maintained on a rotating platform with an orbital diameter of 1.9 cm (Thermo Scientific, Loughborough, UK) at 185 revolutions per minute (rpm) in a humidified incubator (Thermo Scientific) at 8% CO2 and 37 °C in FreeStyle 293 (Life Technologies, Paisley, UK) without serum or antibiotics. Cell growth was monitored by determining live cell density using 0.2% trypan blue dye exclusion and a Neubauer improved hemacytometer (Sigma, Gillingham, UK). Cells were passaged twice a week by diluting to 3.5 × 10^5^ live cells per mL.

### Lentiviral vector production

A third generation HIV-1-based lentiviral vector, expressing an EGFPLux fusion protein under the control of a human cytomegalovirus immediate early enhancer and promoter without intron (CMV-) and pseudotyped with Sendai virus F/HN proteins (rHIV.F/HN CMV- EGFPLux), was produced by transient transfection of HEK 293T cells)^30^. This vector was generated using the HIV-1 vector genome plasmid pGM290 (a derivative of pRRLSIN.cPPT.PGK-GFP.WPRE (Addgene #12252) where the PGK, GFP and WPRE sequences were replaced with the CMV enhancer/promoter from pCIKCFTR^77^, EGFPLux from pEGFP-Luc (Promega), and the mut6 form of the WPRE sequence lacking an intact X protein coding sequence)^78^. Additional HIV-1 and Sendai virus F/HN proteins were supplied by plasmids pGM281 (a monomeric form of pMDLg/pRRE (Addgene #12251), pRSV-Rev (Addgene #12253) as well as pGM301 and pGM303, derivatives of pCAGGS-Fct4 and pCAGGS-SIVct + HN)^13^ respectively, where the ampicillin resistance gene has been substituted for a kanamycin resistance gene. For virus production the plasmids pGM290, pGM281, pRSV-Rev, pGM301 and pGM303 were used in a mass ratio of 20:10:5:7:7 respectively.

A 1000 mL polycarbonate flask (Corning, Flintshire, UK) was seeded with 240 mL of HEK 293T cells at 1 × 10^6^ ± 10% cells/mL. These were transiently transfected with a “transfection mix” of 1.5 µg plasmid DNA per 106 cells using 25 kDa branched polyethylenimine (PEI) (Sigma) at an N:P ratio (ratio of moles of amine groups of cationic polymers to moles of phosphate groups of DNA) of 15:1^79^. Twenty-four hours after transfection, the cells were centrifuged and the supernatant replaced by 225 mL fresh FreeStyle 293 to reduce PEI toxicity. At this point, sodium butyrate (Sigma), a known enhancer of transfection efficiency and transgene expression^80^, as well as of lentiviral vector production^81, 82^, was added at a concentration of 5 mM. The cells were then split into the required number of 125 mL polycarbonate flasks (Corning) containing 18 mL volume in each to evaluate the different supplements. The supplements tested were: Cholesterol Lipid Concentrate (CLC), Chemically Defined Lipid Concentrate (CDLC) (Life Technologies), Lipid Mixture 1 (Sigma), Gelatin Peptone N3, (Organotechnie, La Courneuve, France), N-Acetyl-L-Cysteine (NAC) (Sigma) and Pluronic F-68 (Life Technologies). Each was diluted to the desired concentration in FreeStyle 293, filtered using a 0.2 μm surfactant-free cellulose acetate filter (Thermo Scientific), and 2 mL was added to three replicate flasks. For each experiment, three flasks were not supplemented and had only 2 mL of FreeStyle 293 added to act as the non-supplemented control. After a total incubation of 72 hours, cells were harvested and pelleted by centrifugation, 6 minutes at 500 rcf. The supernatant was filtered using a 0.45 µm filter with a Supor membrane (Pall, Portsmouth, UK) and either used immediately for titration or stored at −80 °C.

### Lentiviral vector titration

HEK 293T cells were seeded at 5 × 10^5^ ± 10% cells per well in a 12-well plate (Corning) in FreeStyle 293 containing 8 µg/mL hexadimethrine bromide (polybrene) (Sigma), a known enhancer of retrovirus transduction efficiency^83^. The cells were subsequently transduced by the addition of diluted virus stocks – typically 1:5 diluted virus in FreeStyle 293, though other dilutions were used as necessary to ensure the proportion of transduced cells did not exceed ~20%. After 72 hours’ incubation, EGFP production was confirmed by fluorescence microscopy and the cells were harvested in 5 mL polystyrene round-bottom tubes (Corning) and centrifuged 6 minutes at 500 rcf. Pellets were resuspended in 350 μL of flow cytometry buffer containing 10% (v/v) Bovine Serum Albumin (BSA), 10 mM EDTA and 2% (v/v) paraformaldehyde (PFA) in Dulbecco’s phosphate-buffered saline (Sigma). The number of EGFP positive cells was determined using a FACSCalibur (BD, Oxford, UK) cell sorter and associated CellQuest Pro 6.0 (BD) software. Titres were obtained by gating for EGFP positive cells compared to triplicate untreated samples average set at ≤0.05% EGFP positive cells, and converting the obtained percentage into flow cytometry transducing units (FTU/mL), a measure of functional titre, by multiplying by the number of cells per well and the sample dilution. For each experiment, titres were normalised with the control condition of each experiment set at 100%.

### Statistical analysis

Where possible, statistically significant differences between group means were determined by one-way ANOVA using Prism 7 (GraphPad, La Jolla, CA, USA) and reported as F(k-1, N-k) = F value, p = p value, where k is the number of groups and N the number of data points. This was followed, if appropriate, by Dunnett’s post-test, to compare each of a number of treatments with a single control. A calculated p value of >0.05 was deemed non-significant (indicated with “ns” on figures). Calculated p values of <0.05 were deemed a significant difference and indicated with one star (*) on figures. Error bars in figures represent the standard error of the mean (SEM). Statistical power was evaluated using G*Power 3.1^84^ (University of Düsseldorf, Düsseldorf, Germany).

### Data Availability

All primary data can be made available upon request.

## Electronic supplementary material

Supplementary  Information
