# Author Correction: Assessment of selected media supplements to improve F/HN lentiviral vector production yields

**DOI:** 10.1038/s41598-018-24608-4

**Published:** 2018-04-25

**Authors:** Jean-François Gélinas, Lee A. Davies, Deborah R. Gill, Stephen C. Hyde

**Affiliations:** 1 Gene Medicine Research Group, NDCLS, Radcliffe Department of Medicine, John Radcliffe Hospital, Oxford University, Oxford, UK; 2 United Kingdom Cystic Fibrosis Gene Therapy Consortium, Oxford, Edinburgh, London, UK

Correction to: *Scientific Reports* 10.1038/s41598-017-07893-3, published online 31 August 2017

This Article contains a typographical error.

In the Methods section under subheading ‘Lentiviral vector production’,

“These were transiently transfected with a “transfection mix” of 1.5 µg plasmid DNA per 106 cells using 25 kDa branched polyethylenimine (PEI) (Sigma) at an N:P ratio (ratio of moles of amine groups of cationic polymers to moles of phosphate groups of DNA) of 15:1”

should read:

“These were transiently transfected with a “transfection mix” of 1.5 µg plasmid DNA per 10^6^ cells using 25 kDa branched polyethylenimine (PEI) (Sigma) at an N:P ratio (ratio of moles of amine groups of cationic polymers to moles of phosphate groups of DNA) of 15:1”

