# Supplementary material for: Assessment of selected media supplements to improve F/HN lentiviral vector production yields
Source: Sci Rep. 2017 Aug 31;7:10198. doi: 10.1038/s41598-017-07893-3 (PMC5579034; doi:10.1038/s41598-017-07893-3)
Supplement: Supplementary file 1 — Supplementary Information [file 41598_2017_7893_MOESM1_ESM.doc]

**Supplementary Information For:**

**Assessment of selected media supplements to improve F/HN lentiviral vector production yields**

Jean-François Gélinas^1^, Lee A Davies^1^, Deborah R Gill^1*^, Stephen C Hyde^1*^

^1^Gene Medicine Research Group, NDCLS, Radcliffe Department of Medicine, John Radcliffe Hospital, Oxford University, Oxford, UK and United Kingdom Cystic Fibrosis Gene Therapy Consortium

* Correspondence: SC Hyde E-mail: steve.hyde@ndcls.ox.ac.uk

**Supplementary Results**

The impact of CDLC addition to rHIV.VSV-g CMV- EGFPLux production prior to transfection was evaluated. Flasks containing 7 x 10^5^ cells/mL in 20 mL media were supplemented CDLC for final dilutions of 1:500, 1:250 or 1:50 of the supplied concentrate. Flasks without CDLC supplementation served as a negative control. Each flask was then incubated for 24 hours. Samples from each of these flasks were selected, after incubation, and 1 x 10^6^ cells were seeded in a 6 well ultra-low attachment plate (Corning) in 2mL of media containing the appropriate dilution of CDLC. These were transfected with 5µg of the plasmid mixture required to produce rHIV.VSV-g CMV- EGFPLux. Twenty-four hours after transfection, sodium butyrate was added to each well at a final concentration of 5mM. A trend for decreased virus production in the presence of CDLC pre-transfection was observed (Figure S1) prompting the use of supplements post-transfection in all other experiments.

**Figure S1. Lentiviral vector titres following lipid medium supplementation pre-transfection.** Yields of rHIV.VSV-g CMV- EGFPLux in non-supplemented medium (dark green) or medium supplemented (yellow) with Chemically Defined Lipid Concentrate added 24 hours before transfection. Titres were determined by flow cytometry and are shown relative to non-supplemented values.
